# Excessive homozygosity identified by chromosomal microarray at a known GCDH mutation locus correlates with brain MRI abnormalities in an infant with glutaric aciduria

**DOI:** 10.1002/ccr3.1054

**Published:** 2017-06-28

**Authors:** Abdul Ali Peer‐Zada, Ali M. Al‐Asmari

**Affiliations:** ^1^ Molecular Pathology (Genetics) Section Pathology and Clinical Laboratory Medicine Administration King Fahad Medical City Riyadh Saudi Arabia; ^2^ Department of Pediatrics Medical Genetics Section King Fahad Medical City Riyadh Saudi Arabia

**Keywords:** Chromosomal microarray, exome sequencing, glutaric aciduria, homozygosity, magnetic resonance imaging, Sanger sequencing

## Abstract

Herein, we report a conceptually novel clinical case highlighting the diagnostic implications of excessive homozygosity and its correlation with brain MRI abnormalities in an infant with GA1. The case also points a need for an extra amount of caution to be exercised when evaluating patients with “negative exomes.”

## Clinical Report

**Figure 1:**
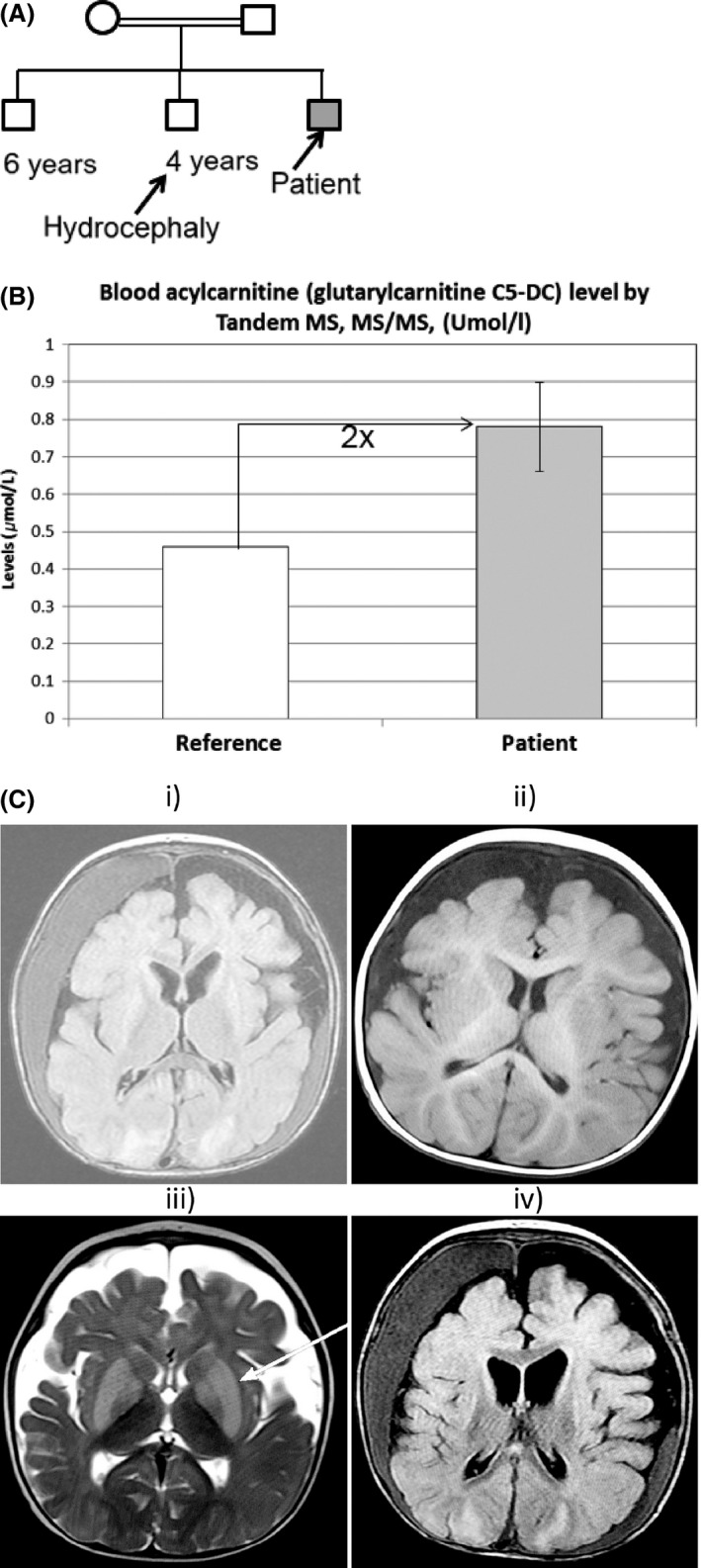
Family structure and biochemical test performed on the affected patient. (A) Pedigree showing consanguineous marriage with the arrows showing the patient (solid box) and his 4‐year‐old brother with hydrocephaly. (B) Glutarylcarnitine (C5‐DC) levels in the blood as measured by Tandem MS, MS/MS in‐house at KFMC. (C) Brain MRI in patient with GA1. Axial T1 (C, i–ii)‐, T2 (C, iii)‐weighted and flair (C, iv) MR images showing atrophy, bilateral subdural collections and enlarged pretemporal subarachnoid spaces and batwing appearance of enlarged Sylvian fissures. Basal ganglia appear small and nuclear signal abnormalities are observed (arrow in iii).

We describe a Saudi male patient who presented at 11 months of age. He is a known case with developmental regression, hepatosplenomegaly, seizure disorder, motor delay, oropharyngeal swallowing problems, and recurrent chest infections, which started after the age of 7 months and was hospitalized. He was intubated twice for 6 weeks and has been on nasogastric tube (NGT) feeding since. The patient is the third male child of a consanguineous marriage with the mother having five miscarriages (Fig. 1A). The second male child has hydrocephaly.

## Initial Physical and Laboratory Examination

The clinical assessment revealed visual localization and tracking, tactile and proprioceptive/vestibular sensory processing, and cognitive abilities as impaired. He had mild hypertonicity on upper limbs, fair head control, and poor trunk control. He was at risk of falls with the Humpty Dumpty score of 13 and was prescribed bilateral elbow gaiters and bilateral antispastic splints. Further physical examination revealed no respiratory distress, no signs of pain, and stable vital signs. Biochemical examination (creatine, plasma Na, K, Ca, albumin, urea etc.) was unremarkable with slightly elevated plasma ammonia level (65 Umol/L; reference range 16–60). However, his serum free T4 and serum TSH remained persistently very high (mean 52.3 pmol/L; reference range 12.0–22.0) and low (0.049 mIU/L; reference range 1.36–8.8) over time, respectively. Tandem mass spectroscopy MS, MS/MS showed increased glutarylcarnitine (C5‐DC, mean 0.79 ± 0.12; reference range ≤0.46 Umol/L) in three measurements, and gas chromatography GCMS showed high excretion of ketone bodies and lactic acid in the urine (Fig. 1B). Monitoring of the levels of antimicrobial drug vancomycin revealed higher peak concentration of the drug than the maximum level, suggesting increased risk of toxicity.

Mother of the patient reported trying oral feeding; however, patient did not accept feeding. Upon the initial bedside assessment, the patient presented with oral dysphagia (absent tongue cupping, poor jaw excursion, and poor labial seal) and signs/symptoms of aspiration (weak cough with sips of water). Thus, NGT feeding was continued and oral stimulation techniques were explained to the mother. A Modified Barium Swallow study (MBSs) was recommended and performed to further evaluate his oropharyngeal swallowing function.

## Modified Barium Swallow Study

The patient was viewed in the lateral plane only and given the following barium‐impregnated consistencies: Thin liquids (via bottle, using regular‐flow nipple and teaspoon), nectar and honey thick liquids (via teaspoon). It confirmed oral dysphagia and mild‐moderate pharyngeal dysphagia. Specifically, the following observations were noted:

Oral Phase: Inadequate bolus acceptance, poor labial sealing with moderately severe anterior loss. Reduced intraoral bolus pressure, severe oral residues, and reduced base of the tongue retraction. Premature oral spillage to the level of pyriform sinuses and piecemeal swallow deglutition were noted.
Pharyngeal Phase: Velopharyngeal closure was slightly inadequate across trials resulting in one episode of nasal regurgitation (with thin liquids via teaspoon). Adequate hyolaryngeal excursion (HLE) movement was noted in anterior and superior planes. Pharyngeal residues were noted.
Penetration/aspiration: Minimal deep penetration postswallow (with residues) was noted with thin liquids (via bottle and teaspoon) and honey thick liquids (via teaspoon).

Based on the MBSs findings, patient was considered at risk of aspiration and specific precautions such as “seating in an upright position during and 30 min postfeeding, slow rate of feeding, and jaw support.” However, oral feeding was considered safe for thin liquids (via bottle), nectar thick liquids (via teaspoon), and thin puree.

## Radiological Examination

Initial radiological examination revealed enlargement of the heart, no major atelectasis or consolidation of the lungs and no pleural effusion or pneumothorax. Ultrasonography showed normal size liver measuring 9.5 cm in craniocaudal dimension with persistent mildly echogenic and heterogeneous echotexture of the parenchyma. No focal lesion was seen. The portal and hepatic veins and IVC are patent. No intrahepatic or extrahepatic biliary duct dilatation was seen.

The gallbladder was observed as contracted, and the visible pancreas showed no lesion. Due to fluid‐filled distended stomach, the spleen was partially visualized. Both kidneys were normal in size, shape, and location. The right kidney measured 6.2 cm and the left kidney 6.1 cm. The parenchymal echogenicity was normal with preserved corticomedullary differentiation. No pelvicaliectasis, cyst, and calculi were seen. Floating debris within the urinary bladder was noted. There was no free fluid or localized fluid collection seen in the abdomen and pelvis.

## Brain MRI

**Figure 2:**
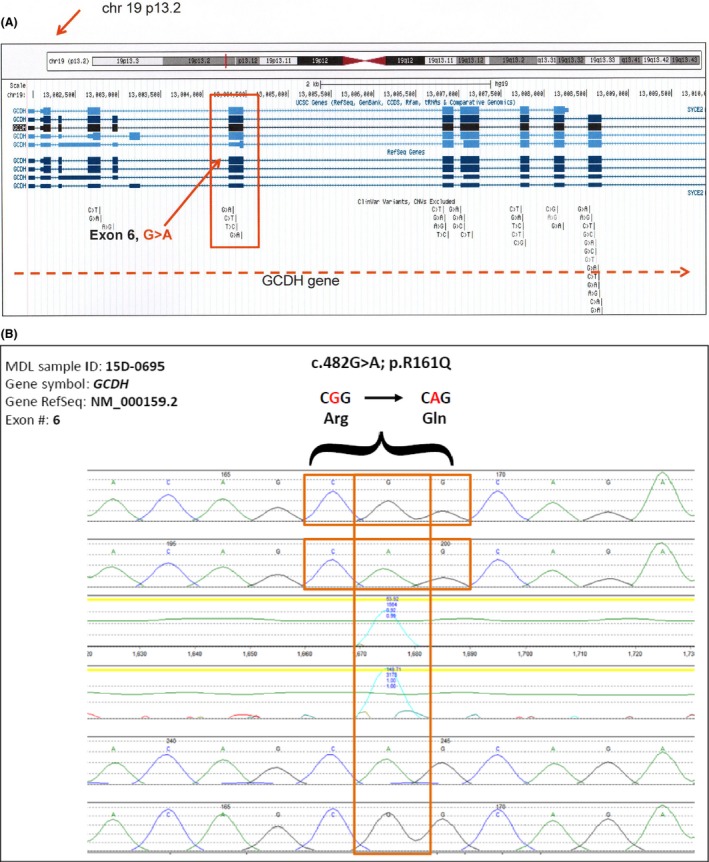
Molecular genetic analysis of the affected patient. (A) UCSC genome browser derived picture showing GCDH gene at the chromosomal location corresponding to the excessive homozygosity from CMA report shown in (A). (B) Sanger sequencing of exon 6 of the GCDH gene showing homozygous missense mutation (boxed).

MRI revealed bilateral subdural collections with mass effect on the brain parenchyma, more in the right side (Fig. 2C). The signal intensity of collection is bright on T2‐weighted images; in T1‐weighted images, some areas demonstrated signal intensity higher than CSF fluid. Both occipital lobes demonstrated cortical and subcortical high signal intensities in T2‐weighted images and with linear high signal intensities in T1‐weighted images. There was evidence of resolving basal ganglia restricted diffusion with atrophy. Findings suggested old hypoxic ischemic brain injury, but the constellations of findings suggested glutaric acidemia or aciduria (GA1).

## Molecular Genetic Analyses

Owing to heterogeneous clinical phenotype compounded by consanguinity, family history, and recurrent miscarriages in the family, genomic technologies viz, chromosomal microarray using both copy number and single‐nucleotide polymorphism probes on a whole genome array (Affymetrix Cytoscan HD platform, Mayo Clinic) and exome sequencing with exome enrichment (Agilent SureSelectXT Human All Exon 50 Mb) on Illumina HiSeq (BGI‐Europe, Denmark) were requested. Targeted Sanger sequencing for mutation in glutaryl‐CoA dehydrogenase gene (GCDH, OMIM 608801) that is known to cause GA type 1 (GA1, OMIM 231670) 1 was used for confirmation (King Faisal Specialist Hospital and Research Center Laboratory, Saudi Arabia).

CMA revealed no significant copy number changes in any specific diagnostic condition but identified several regions of homozygosity (3 Mb or larger), encompassing at least 9% of the genome, raising the possibility of an autosomal recessive disorder due to the presence of a homozygous mutation in one of these regions. The genetic location for the region of homozygosity was chr19:4822854‐20498306, 19p13.3‐p12 15,675. Interestingly, the region of excessive homozygosity carries the GCDH gene known to be mutated in GA1. Targeted sequencing revealed a homozygous variant c.482G>A of missense mutation p.R161Q in exon 6 of the GCDH gene confirming the diagnosis of GA1 in support of MRI findings.

Exome sequencing did not reveal any specific causative pathogenic mutations in the genes associated with mitochondrial or with metabolic disease.

## Discussion

GA1 is an autosomal recessive metabolic disorder caused by a missense mutation in a flavin adenine dinucleotide (FAD)‐requiring mitochondrial enzyme GCDH that leads to the deficiency of the enzyme activity 1. The enzyme GCDH, which is active in mitochondria as a homotetramer, catalyzes the dehydrogenation and subsequent decarboxylation of glutaryl‐CoA, a catabolite of L‐lysine, L‐hydroxylysine, and L‐tryptophan to glutaconyl‐CoA and crotonyl‐CoA, respectively 2. Deficiency of GCDH enzyme activity characteristic of GA1 and GA2 leads to the accumulation of glutaric acid, 3‐hydroxyglutaric acid, and glutaconic acid in the blood, urine and CSF and brain tissue that may in turn induce imbalances in neurotransmission and produce neurotoxic effect 3.

Clinically, GA1 in children, like the case being described in the current study usually presents with macrocephaly at, or shortly after birth after a normal initial development. Neurological abnormalities in the first episode appear between 6 and 18 months of age, often triggered by febrile infections. Our patient presented with recurrent chest infections, neurodegeneration following a metabolic stress at 10 months of age. Hypoxic ischemic brain injury, bilateral subdural collections with mass effect on the brain parenchyma, atrophy in the patient were indicative of a poorer prognosis. However, since age at onset predicts severity of motor impairment and clinical outcome of GA1 4, early definitive diagnosis of GA1 in the patient is therefore essential. MRI of the brain is one of the main modalities to investigate children with possible GA1. It shows atrophy or hypoplasia of the frontotemporal regions of the cerebral hemispheres, enlarged pretemporal middle cranial fossa subarachnoid spaces, and cyst‐like dilatation of the Sylvian fissures with “batwing” or “box‐like” fissures 5. Additional findings include hyperintense signal on T2‐weighted images involving the dentate nuclei and cerebral hemispheric white matter. As the disease progresses, generalized cerebral atrophy, ventricular dilatation, and basal ganglia atrophy accompanied by subdural hemorrhages become more conspicuous. Edema within the putamen and caudate are manifest by increased signal intensity on T2‐weighted imaging 6. Our patient presented with MRI brain features suggestive of GA1.

Molecular genetic testing is used not only to confirm clinical diagnoses in newborns undergoing inpatient and outpatient care but also used for second‐tier confirmation and sometimes for rapid diagnosis. In this setting, genomic technologies such as CMA and WES are increasingly being utilized due to affordability, reduced costs, and above all providing a higher diagnostic yield molecular genetic testing is used not only to confirm clinical diagnoses in newborns undergoing inpatient and outpatient care but also used for second‐tier confirmation and sometimes for rapid diagnosis. In this setting, genomic technologies such as CMA and whole exome sequencing are increasingly being for disorders with suspected genetic etiology 7, 8, 9. Exome sequencing in Saudi pediatric genetic disorders (Peer Zada et al., in Review) has also revealed a diagnostic yield of about 48%. The mutation detection rate increased to about 60% when consanguinity and family history alone were taken into account, suggesting the two factors as main indications for genomic testing. Genomic technologies have also been proposed for newborn and pediatric diagnostic medical care 10. The excessive homozygosity identified by CMA analysis in our patient indicating a possible autosomal recessive disorder could be attributed to consanguinity in this family. The incidence of GA1 has been reported to be more among heavily consanguineous communities, such as the Amish, and the Indians in Canada 11, 12. Saudi Arabia has a reported consanguinity rate of about 50–60% that may reach 80% in some tribal areas 13, 14. Detailed analyses of the chromosomal region of excessive homozygosity in the “genome browser” revealed the presence of GCDH gene in this region (chromosome 19p13 locus) that is known to be mutated in GA1. GCDH gene maps to chromosome 19p13.2 and consists of 12 exons encoding 438 amino acid protein (NM_000159.2; NP_000150.1). Testing for mutations in the coding regions and splice junctions in the GCDH gene using PCR and Sanger sequencing technologies revealed a reported homozygous missense mutation (c.482G>A; p. R161Q). Taken together, the region of excessive homozygosity by CMA containing the GCDH gene mutation as confirmed by sequencing correlates with MRI brain results in this particular case.

An important question that arises here is whether multiplatform strategy as adopted in this clinical case was actually necessary and effective in the diagnostic workup of the patient. We believe that this in fact is the case given the phenotypic heterogeneity of the patient who presented with chest infection and an overlap with other diseases such as failure to thrive, developmental, motor and speech delay, seizures in addition to being a case of a consanguineous marriage with the mother having five miscarriages and strong family history (Fig. 1A). In the case of GA1 even, where the clinical diagnosis points to well‐known gene genotyping heterogeneity does exist, for example, 63 different types of mutations known in GCDH1 15, 16. Additionally, in some countries including Saudi Arabia where all clinical and/or genetic tests are not available and have to be sent to referral laboratories outside the country with a longer turn‐around‐time, it would be more pragmatic for the clinical geneticist to decide on a panel of molecular genetic tests.

The exome sequencing in our patient did not reveal any causative pathogenic mutations (false‐negative result). We presume that the mutation in the GCDH gene may have been missed due to some challenges that still exist in exome capture technologies as well as the variant calling algorithms in the data analysis process of exome sequencing. Despite this known technological limitation, effective communication between the clinicians and the laboratorians would be essential particularly when the clinical phenotype provides a strong suspicion for a responsible genotype and the results are negative such as in our case. In the case of “negative exomes” the questions that a physician needs to raise are as follows: is the gene or the locus he is looking for covered by the exome panel? If so, is there poor coverage over that region?

In conclusion, we show for the first time a conceptually novel correlation between excessive homozygosity by CMA and MRI brain abnormalities in GA1a in a clinical case from Saudi Arabia. Second, a multiplatform strategy may be effective in the diagnostic and/or prognostic workup of patients with phenotypic and genetic heterogeneity, particularly in the countries where all the clinical tests are not available. Third and importantly, there is a need for an extra amount of caution to be exercised when evaluating patients with “negative exomes” in terms of addressing some important questions pertaining to exome data with the laboratory in such situations so that alternative patient management strategies are applied.

## Conflict of Interest

The authors have no conflict of interest disclosure.

## Authorship

AAP‐Z: performed analyses of all the laboratory results, involved in conceptual design, and writing and editing of the manuscript. AMA‐A: was involved in the clinical management of the patient, performed all the clinical investigations and involved in writing of the clinical part of the manuscript.
